# Dose reduction in CT imaging for facial bone trauma in adults: A narrative literature review

**DOI:** 10.1002/jmrs.319

**Published:** 2019-02-01

**Authors:** Tayla Hooper, Grace Eccles, Talia Milliken, Josephine R. Mathieu‐Burry, Warren Reed

**Affiliations:** ^1^ Discipline of Medical Radiation Sciences the University of Sydney Lidcombe Australia

**Keywords:** Computed tomography, cone‐beam, dose reduction, dual‐source, facial bone trauma, intraoperative

## Abstract

Trauma to the facial area accounts for a significant number of admissions to the emergency department. Diagnostic imaging is almost always required, and is critical in determining patient management. Multi‐detector computed tomography (MDCT) appears consistently in the literature as the gold‐standard imaging modality for facial bones, but results in a high radiation dose to the patient. This makes the application and advancement of dose reduction and dose optimisation methods vital. This narrative review presents a critical analysis of the literature concerning diagnostic imaging of facial bone trauma, with an emphasis on dose reduction methods for MDCT. Databases including Pubmed, Medline, Web of Science and Scopus were used to investigate this topic, with the key words: facial bone trauma, computed tomography (CT) imaging and dose reduction. Exclusion criteria included studies on nasal bone fracturing, dental imaging, elective surgeries and paediatric imaging. The literature shows overwhelming support for MDCT, given its accuracy, efficiency and ease of operation. Noise reducing reconstruction algorithms show promise as a successful method of dose reduction in facial bone imaging. Investigations of more innovative techniques also appear within the literature, including diagnostic cone‐beam CT (CBCT), intraoperative CBCT and dual‐source CT (DSCT), but further research is required to confirm their clinical value.

## Introduction

Diagnostic imaging plays a major role in the management of trauma patients. The efficient and accurate assessment of injuries can optimise treatment to improve patient outcomes. Trauma to the facial area accounts for a large number of admissions into the emergency department with epidemiological studies showing a steady increase in recent years.^1^, ^2^, ^3^, ^4^, ^5^, ^6^, ^7^, ^8^, ^9^ Facial trauma is associated with a high level of morbidity and mortality. This can be due to compromise of the intricate bony structure or vasculature, or it can be attributed to the effects of concomitant pathology and complications, where most patients are polytrauma cases.^5^, ^6^, ^7^, ^10^, ^11^, ^12^, ^76^

All facial bone traumas are initially treated as a medical emergency and there is a demand for timely diagnosis. Subsequent to a primary examination, diagnostic imaging is requested to assess the extent of damage.^1^, ^6^, ^12^, ^13^ Within the current literature, multi‐detector CT (MDCT) is presented as the gold‐standard imaging modality for the diagnosis and management of complex facial trauma in adult patients.^1^, ^4^, ^9^, ^11^, ^15^, ^16^, ^17^, ^18^, ^19^, ^21^, ^22^, ^23^, ^24^, ^25^, ^26^, ^27^ MDCT has superseded the use of plain radiography as first‐line imaging because of the greater diagnostic accuracy, the speed of image acquisition and the capacity to scan polytrauma patients or patients with a reduced Glasgow Coma Scale.^1^, ^6^, ^11^, ^16^, ^17^, ^18^, ^19^, ^21^, ^22^, ^23^, ^24^, ^25^, ^26^, ^27^, ^55^, ^58^

**Figure 1:**
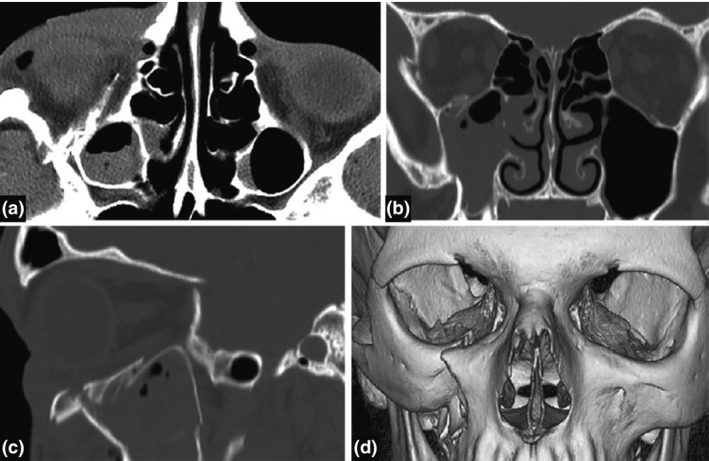
The intricate facial anatomy as seen using multiplanar reconstruction and 3D imaging of the same acquired data: (A) axial slice of the ethmoidal air cells, (B) coronal slice of the osteomeatal complex, (C) sagittal slice of the right orbit, (D) 3D reconstruction of the maxillofacial anatomy.^70^ Permission was obtained to reproduce these images.

MDCT is reported to accurately identify all bony injuries, foreign bodies and concomitant soft tissue pathology for >95% of cases. Fractures missed on MDCT are almost always described as clinically insignificant.^6^, ^16^, ^27^, ^76^ The diagnostic precision of MDCT is supported by the ability to manipulate and reconstruct image data. Image processing, artefact suppression of dental amalgam, multi‐planar reconstructions and 3D imaging, can be used to optimise spatial and contrast resolution and provide a comprehensive view of the facial anatomy (Fig. 1).^4^, ^11^, ^12^, ^16^, ^22^, ^26^, ^27^, ^28^, ^29^, ^30^, ^44^, ^76^, ^79^ This technology also allows for treatment planning and post‐operative assessments.^23^, ^24^, ^25^, ^26^, ^31^, ^32^, ^33^, ^34^, ^35^, ^75^, ^79^ 3D models from pre‐operative scans facilitate personalised surgical planning including the design of custom reconstructive metal plates.^24^, ^26^, ^31^, ^34^, ^35^, ^75^, ^79^

Despite the advantages of MDCT in imaging adult facial trauma patients, the associated radiation dose remains a concern because the effective dose (ED) exceeds that of plain radiography. The average ED for OPG is 0.014 mSv, and ED can range from 0.02–0.1 mSv for plain x‐ray of the skull or face. By comparison, the estimated ED from facial bone MDCT varies from 1 to 5 mSv^2^. Recent publications have focused on methods for dose reduction in MDCT. A number of techniques have emerged as the possible direction for imaging facial bone trauma; however, an inclusive analysis has not yet been published. The purpose of this review was to compare literature on these approaches so technicians and future research groups can have insight on how dose issues may be addressed in facial bone CT.

## Methods

A narrative literature review was undertaken to allow for comprehensive coverage of published qualitative and quantitative data.^14^ The primary focus of the review was on dose reduction for CT in imaging of complex facial bone trauma in adult patients. Recent literature was utilised to relate results to the current evidence‐based practice. This review was limited to articles published in English. Furthermore, because the research was conducted as a narrative review instead of systematic, the results are largely descriptive and exploratory.

**Figure 2:**
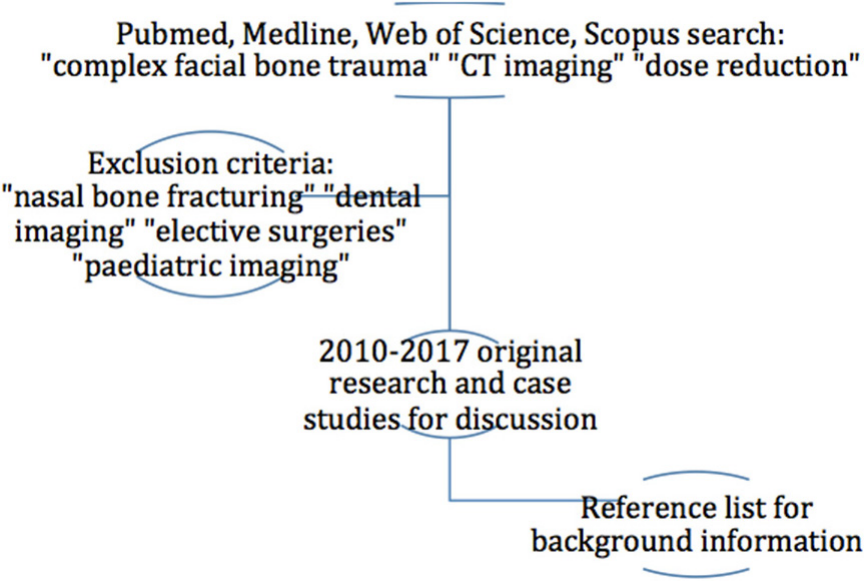
The selection process for articles included in the results.

PubMed, Medline, Web of Science and Scopus databases were searched using the key MeSH terms “complex facial bone trauma”, “CT imaging”, and “dose reduction”. Chosen articles were original research and case studies with publication dates from 2010 to 2017. Earlier articles were sourced from the reference list of these studies to provide an overview on dose reduction development over time. Articles that focused on the diagnostic efficacy of MDCT alone were used for background information. Exclusion criteria included studies on nasal bone fracturing, dental imaging, elective surgeries and paediatric imaging. A total of 43 articles were selected for inclusion in the literature review (Fig. 2). Of these, 41 articles were original research and two were case studies. An additional review article was included to help establish the dose from MDCT.

## Discussion

The consensus across the literature is that MDCT is the imaging modality of choice for adult patients with complex facial bone trauma.^17^, ^28^, ^37^, ^38^ The liberal use of MDCT has become a concern because the ED exceeds that of plain radiography at an average of 1.5–2.9 mSv per facial bone CT compared to 0.02–0.1 mSv for x‐ray.^2^, ^39^, ^40^ The accumulative dose from facial bone trauma would increase over time due to ongoing imaging through the treatment and follow‐up phases.^35^, ^43^ The substantial ED attributed to MDCT warrants ongoing research to optimise dose and prevent reversal of the risk‐benefit ratio.^17^, ^28^, ^37^, ^38^ The dose to patients from MDCT depends upon the technique factors and protocols used; examples of average exposures are shown in Table 1.^34^, ^37^, ^39^, ^40^, ^41^, ^42^, ^43^

**Table 1:** The average dose from facial bone CT for various acquisition and reconstruction methods

Modality	Dose
Conventional MDCT head	2.6–4.0 mSv
Conventional MDCT facial bones	0.9–3.48 mSv
High resolution ultra‐low dose craniofacial MDCT – filtered back projection	0.9–3.6 mSv
High resolution ultra‐low dose craniofacial MDCT – adaptive statistical iterative reconstruction	0.82–2.19 mGy
High resolution ultra‐low dose craniofacial MDCT – model‐ based iterative reconstruction	0.22–0.82 mGy
Cone beam CT – facial bones	0.35 mGy
Intraoperative MDCT – facial bones	0.25–3.6 mGy
Intraoperative C‐arm CBCT – facial bones	0.41 mGy
Dual‐source CT with an iterative reconstruction – temporal bone	1.54 mGy

In response to the radiation burden of facial bone MDCT scans, a number of dose optimisation measures have been proposed. Potential solutions have continued to emerge over time as limitations are recognised.^15^, ^28^, ^37^, ^38^, ^46^, ^63^ Trending research has examined the use of protocol changes in MDCT, as well as novel methods including the use of cone‐beam CT (CBCT), intraoperative CT, and dual‐source CT (DSCT) systems. A summary of the primary methods of dose reduction that have been prominent in the literature is outlined in Table 2.

**Table 2:** The primary methods for dose optimisation in facial bone imaging using computed tomography

Method	Evidence of dose reduction	Limitations
Reduction of tube current (mAs)	Reducing mAs from 100 to 40 demonstrated a 55.4% and 38.8% to the lens of the eye and the thyroid respectively.^15^, ^18^, ^19^	Decreased low contrast resolution. Evidence only focused on sinus imaging and is largely out‐dated. May be incorporated in low dose CT protocols.
Low dose MDCT with adaptive statistical iterative reconstruction algorithm	76% dose reduction in craniofacial imaging with significant reductions in noise. Image quality superior to FBP.^37^	Limited efficacy in detecting non‐displaced fractures due to smoothing effects.
Low dose MDCT with model‐based iterative reconstruction algorithm	91% dose reduction in craniofacial imaging, with superior reduction in noise. Image quality superior to FBP and ASIR.^37^	No bone kernel reconstruction at the time of writing. Bone smoothing effects could limit diagnosis.
Elimination of dedicated facial bone CT	Screening via head CT is specific and sensitive in the detection of mid‐face fractures.^28^	Evidence only concerned with blunt trauma, small sample size. No significant dose reduction.
Cone beam CT	Lower tube current and single rotation of x‐ray source. Effective in detecting orbital floor and zygomaticomaxillary fractures.^20^, ^38^	Poor contrast resolution. Not appropriate when trauma of the cervical spine is suspected because patient movement is required.
Intraoperative CT	Reduces the need for pre‐ and post‐operative scans. Can be used in conjunction with CBCT to reduce dose^59^, ^60^, ^61^, ^62^, ^63^, ^64^, ^65^	Limited research into the dose associated with intraoperative MDCT and CBCT.
Dual‐source CT	Increase in pitch and independent x‐ray sources reduce dose. New generations have improved image quality.^42^, ^66^, ^67^, ^68^	Limited research into assessment of the entire facial structure with this technology.

### Protocol‐based dose reduction in MDCT

Earlier literature advocated for the reduction of exposure factors as an effective method of dose reduction in facial bone MDCT. Studies demonstrated significant reductions in dose without compromise of diagnostic image quality.^46^, ^47^

Lorenzen et al.^46^ reported that a reduction of tube current from 150 to 30 mAs reduced ED by 70% during examinations of a phantom mid‐face. The results of these exploratory studies formed the foundation of low dose and ultra‐low dose MDCT protocols trending in the current literature.

#### Low dose and ultra‐low dose protocols with filtered back projection

There are various low dose and ultra‐low dose protocols, which are derived from reductions in tube current. Early investigations of this technique^48^, ^49^ focused on modifying protocols for facial sinus imaging. These studies demonstrated that a significant decrease in mAs facilitated an 8‐fold reduction in ED. These results were promising, however, because imaging after facial trauma necessitates clear examination of soft tissue structures as well as bone, these protocols could not be generalised.

More recent studies suggest pairing low dose and ultra‐low dose MDCT protocols with novel reconstruction algorithms, to reduce dose while maintaining diagnostic image quality.^15^, ^18^, ^19^ Since the inception of MDCT, images have been reconstructed using filtered back projection (FBP). This approach relies on high mAs to combat noise causing absorbed dose to be within the range of 3.48 and 30.48 mGy^15^, ^51^, ^52^ for facial bone MDCT. Adaptive statistical iterative reconstruction (ASIR) and model‐based iterative reconstruction (MBIR) are two of the most prominent reconstruction algorithms in the literature.^15^, ^18^, ^19^

#### Low dose and ultra‐low dose protocols with ASIR and MBIR algorithms

**Figure 3:**
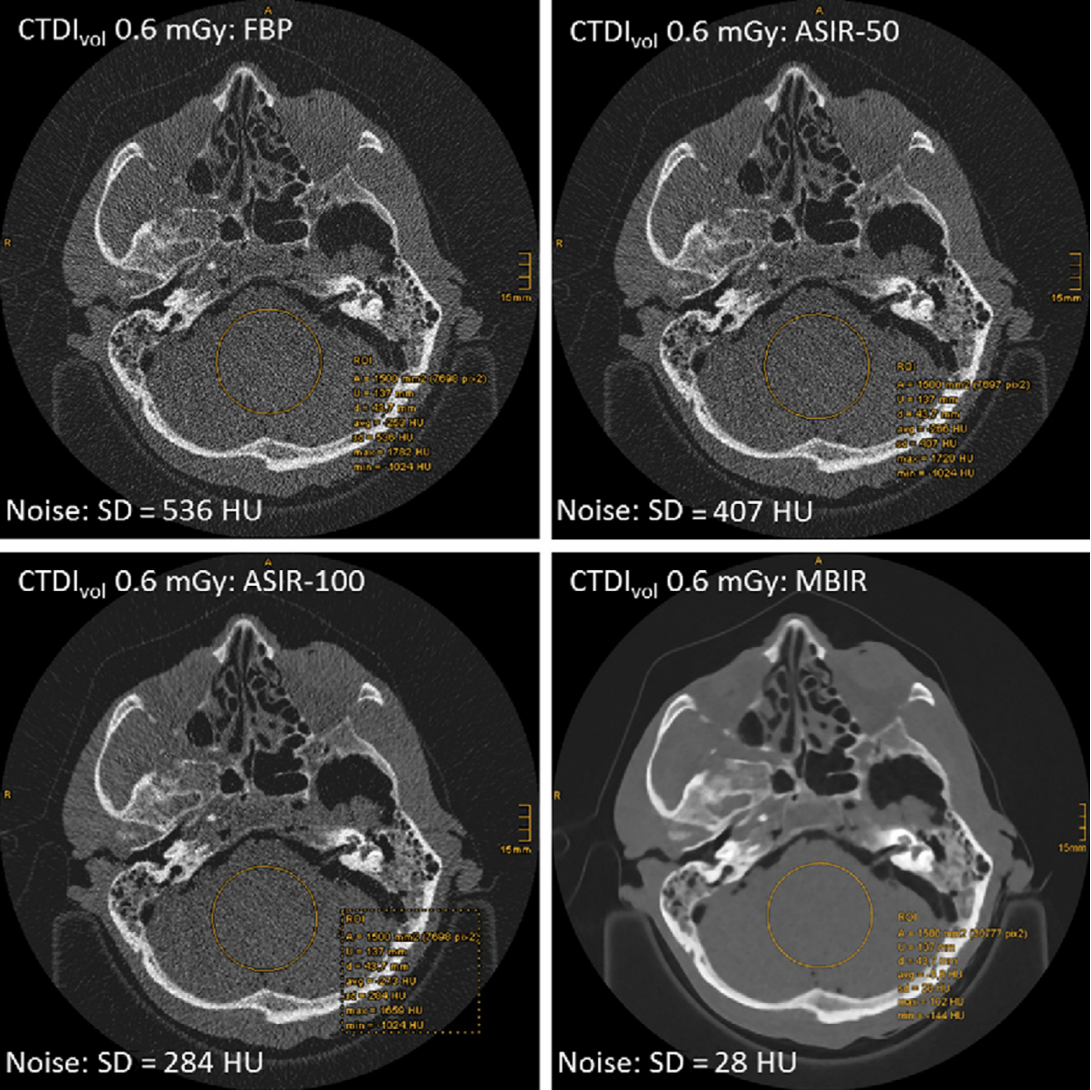
Noise measurement (SD) on axial slices of the mid‐facial region completed on CT with the lowest dose protocol. Results show the lowest noise with MBIR compared to FBP, ASIR‐50 and ASIR‐100 reconstructions.^37^ Permission was obtained to reproduce these images.

ASIR uses information obtained from the FBP algorithm as a scaffold for image reconstruction,^19^ while MBIR incorporates a number of key parameters.^18^ These iterative reconstruction (IR) algorithms are both reported as capable of reducing dose in facial bone imaging, while maintaining low noise and enhancing spatial and contrast resolution.^15^, ^18^, ^19^, ^51^, ^61^ Studies published by Widmann et al^37^, ^61^ demonstrated that the implementation of adaptive statistical IR and model‐based IR in craniofacial bone imaging reduced dose by 76% and 91% respectively. The absorbed dose using adaptive statistical IR can be as low as 2.19 mGy, while for model‐based IR dose can be minimised to 0.22 mGy. A follow‐on study^15^ reported that the use of IR significantly improved subjective image quality for the purpose of navigated surgery on craniofacial fractures, in comparison to images produced through FBP, as shown in Figure 3. On analysis of the results, Widmann et al^37^, ^61^ concluded that although IR reduces noise, further research is necessary to establish whether diagnosis of non‐displaced fractures would become compromised due to the integral smoothing effects.

#### Elimination of dedicated facial bone scans

Another protocol‐based approach to dose reduction in the literature is the elimination of dedicated facial bone MDCT scans.^28^, ^53^, ^78^ It was proposed that head CT data performed as a part of initial trauma imaging, could be reconstructed using soft tissue and bone algorithms to adequately show the facial structures without rescanning the patient. This method has failed to become prevalent because studies have not demonstrated the same diagnostic accuracy with a significant reduction in dose.^28^, ^78^ Although an overlapping scan range is avoided, Lee et al^28^ reported a similar ED for patients examined with a single head MDCT scan and those who underwent separate facial and brain scans. This could be attributed to the need for higher exposure factors for the head CT so high quality facial reconstructions can be produced. A study conducted by Huang et al^78^ concluded that over 80% of patients with a face injury required further dedicated imaging.

#### Elimination of repeat MDCT facial scans

A study published in 2014 by Schmutz et al^36^ supported the use of magnetic resonance imaging (MRI) as an alternative to MDCT for 3D reconstruction and surgical planning for orbital fractures. The use of MRI for facial trauma would be ideal to prevent exposure to this radiosensitive area, however it could not be used as the initial imaging modality unless the presence of metal foreign objects can be confidently excluded. MRI could be used if additional pre‐operative imaging is required, provided that the long scan time is not a concern and there are no contraindications.^9^, ^36^, ^73^ The need for additional imaging following a facial trauma CT is typically negated by the reconstruction and 3D modelling capabilities of MDCT, which allow for comprehensive assessment of the face including the intricate orbital region.^73^, ^79^

### Cone‐beam CT and facial bone imaging

Previously, CBCT has been used almost exclusively in dental radiology. The application of adapted modern systems has been presented as a feasible method to assess the facial anatomy following traumatic injury. CBCT is known to deliver significantly less radiation dose than MDCT, up to 22 times. This is because of the lower tube current and the smaller scan field of view due to the beam shape integral to this technology.^38^, ^54^, ^55^, ^56^, ^57^, ^71^, ^74^, ^77^

**Figure 4:**
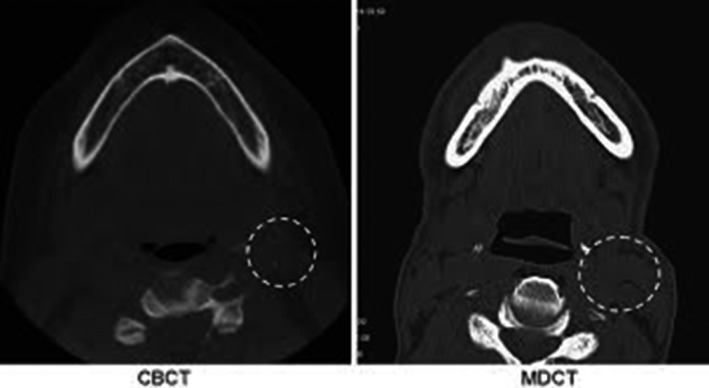
Axial slices through the inferior mandible with CBCT and MDCT shows a loss of soft tissue contrast in CBCT compared to MDCT in the sternocleomastoid region.^38^ Permission was obtained to reproduce these images.

Comparative studies of CBCT and MDCT showed that diagnostic acceptability of images could be maintained with the use of this technology.^20^, ^38^, ^56^, ^71^, ^77^ Brisco et al,^38^ Lezhnev et al^20^ and Veldhoen et al^77^ all advocate for the use of CBCT because the rate of fracture detection in the maxillofacial structures is the same, but the dose is reduced, particularly to the orbital area. However, it was identified that CBCT has limited ability to assess for comorbidities around the bony anatomy because of lower contrast resolution in soft tissue areas,^20^, ^38^, ^77^ as shown in Figure 4. This reduced soft tissue delineation with CBCT limits the use of this modality in post‐operative imaging. Because there is less soft tissue data the effect of artefact suppression is minimised, consequently any metal creates a greater artefact than it would on MDCT scans.^45^ Based on these results, CBCT could only be recommended as an alternative to MDCT when dose is a major concern.^20^, ^38^

In addition to having suboptimal contrast resolution, CBCT is also inferior to MDCT for facial trauma because current imaging units require the patient to be erect. This makes application suboptimal for polytrauma patients that may have simultaneous spinal injury, and any patient with a reduced Glasgow Coma Scale or inability to stand.^55^, ^58^ Although the current published literature is largely descriptive, the significant reduction in radiation exposure and the ease of operation afforded by CBCT imaging systems suggest further research into this modality should be undertaken.

### Intraoperative CT for facial bone surgery

Improving imaging during the treatment of facial bone pathology has emerged in the literature as a way to decrease accumulative patient dose. The application of intraoperative CT has been shown to reduce the number of MDCT scans during the treatment and recovery phases by eliminating the need for pre‐operative and post‐operative scans. The use of intraoperative CT is supported in the literature for the repair of complex facial fractures.^35^, ^43^, ^59^, ^60^, ^62^, ^63^, ^64^ Intraoperative imaging provides real‐time feedback on the facial anatomy during surgery. The use of this technology enables immediate assessment of reduction so minute corrections can be made. A number of studies conclude that the impact of intraoperative CT is significant, with surgical decisions affected in up to 2/3 of patients.^21^, ^60^, ^63^, ^64^ Consolidation of optimal fracture reduction limits the need for additional corrective procedures and subsequent repeat CT imaging.^43^, ^59^, ^60^, ^62^, ^63^, ^64^, ^65^ There is limited literature on dose with the use of intraoperative MDCT and a more comprehensive investigation would be beneficial. Studies by Stuck et al^62^ and Klatt et al^65^ have reported a comparable ED to pre‐operative MDCT examinations.

#### Intraoperative CBCT to reduce ED

Recently, investigations of the use of intraoperative C‐arm CBCT have emerged. The C‐arm configuration of the CBCT enables the patient to be scanned supine during the procedure. Within the literature, this modality is reported as most likely to reduce surgical complication and the need for further imaging while maintaining lower ED.^35^, ^59^, ^60^, ^65^ The radiation dose from intraoperative CBCT is significantly lower than that of MDCT, but the image quality is still appropriate to facilitate surgical assessment and modification. Intraoperative CBCT is therefore proposed as a viable alternative to conventional pre‐operative CT imaging to promote better patient outcomes at lower radiation doses.^59^, ^60^, ^63^, ^65^ The literature has primarily focused on establishing the diagnostic value of intraoperative CBCT and reducing dose by avoiding repeat imaging and surgical correction. A greater investigation into the numerical dose levels should be completed before implementation of this approach to facial bone treatment.

### Dual‐source CT: a possible direction for the future?

**Figure 5:**
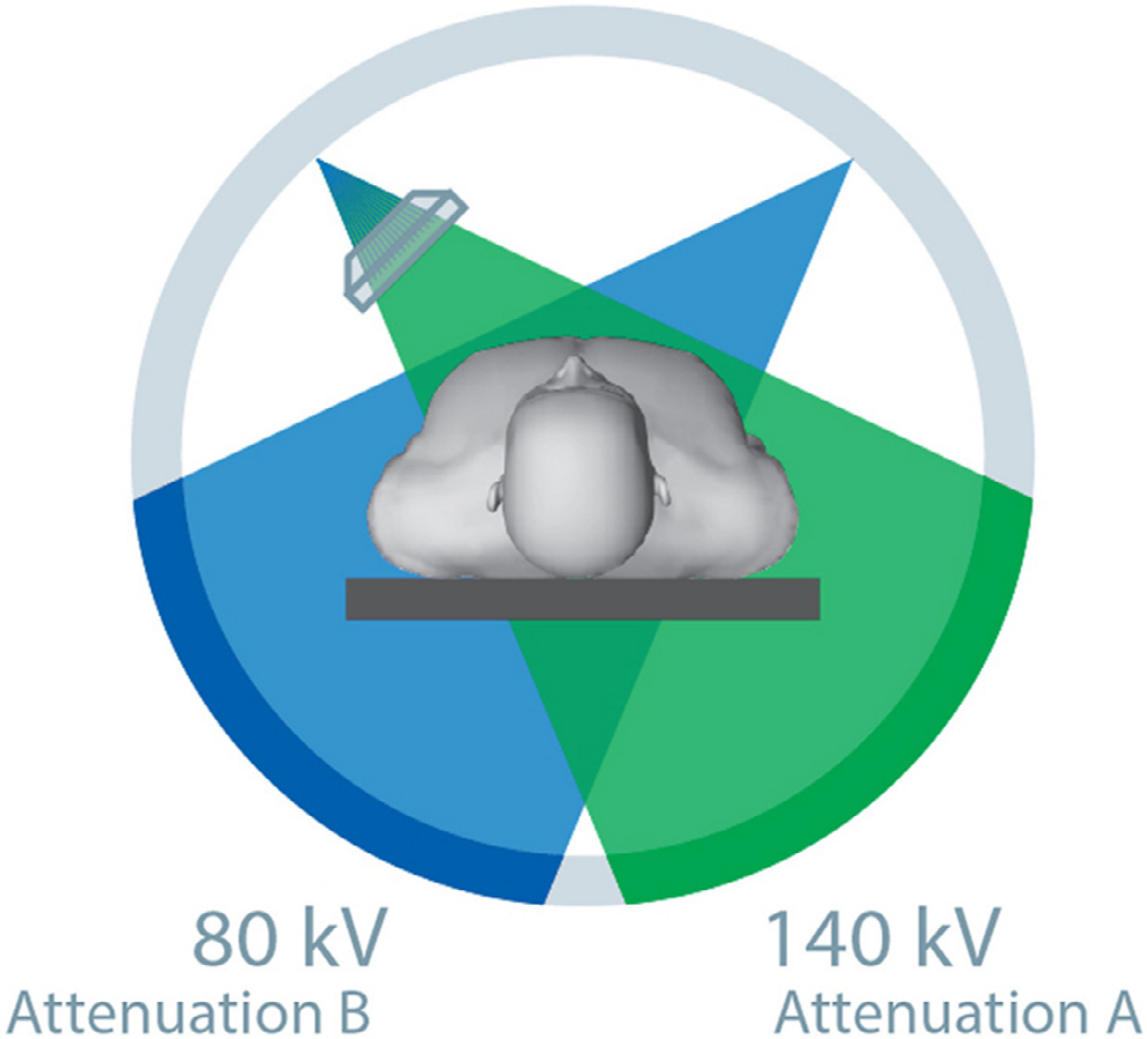
A DSCT system with two x‐ray sources allows two different kVp and mAs settings to be used simultaneously with different amounts of x‐ray attenuation measured depending on the exposure factors.^72^ Permission was obtained to reproduce this image.

Current research into the use of new generation DSCT has revealed this modality as a promising method to reduce patient dose in traumatic facial bone imaging.^42^, ^50^, ^66^, ^67^, ^68^ DSCT systems operate with two independent x‐ray sources that provide continuous, non‐overlapped anatomical coverage (Fig. 5). Dose optimisation is attributed to the increase in pitch and slices acquired per rotation, in addition to the ability to use different exposure and technique factors for each x‐ray source. Dual‐energy CT (DECT) is noted in the literature as the most effective protocol for DSCT to reduce dose while obtaining the most information.^50^, ^66^, ^67^ DECT acquires data using two different energy levels and utilises the attenuation differences to extrapolate more information and apply artefact suppression.^67^ The alternate protocol, high‐pitch CT (HPCT) uses a greater pitch factor to reduce rotation time.^68^ This can yield a lower ED than DECT, however less information is acquired.^66^

**Figure 6:**
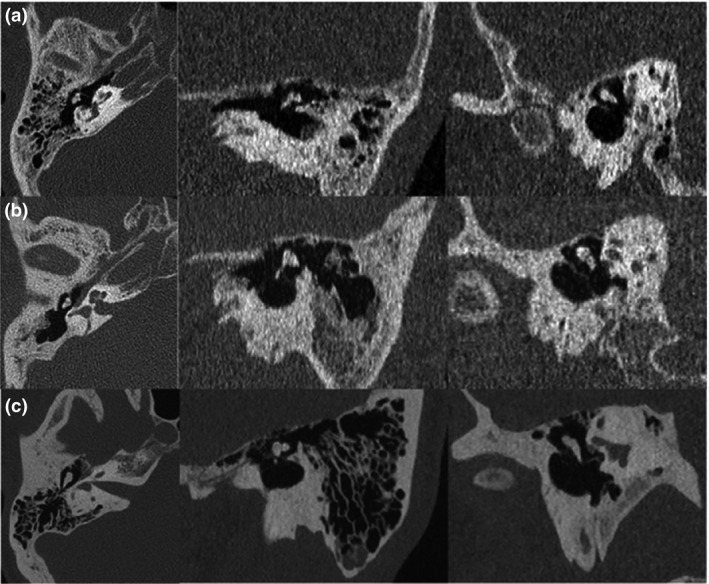
The image quality of temporal bone CT images produced with multiplanar reconstruction using different generations of DSCT scanners, shows the third generation scanners can produce better image quality using lower current to reduce effective dose: (A) first generation images using 180 mAs with ED of 0.67 mSv, (B) second generation slice using 138 mAs with ED of 0.41 mSv, (C) third generation axial slice with 103 mAs and ED of 0.24 mSv.^42^ Permission was obtained to reproduce these images.

Studies on early DSCT generations demonstrated a lesser radiation dose than MDCT, with equivalent or lesser image quality.^66^, ^68^, ^69^ The 3rd generation of DSCT was produced with no *z*‐axis filter and novel IR technology, which promoted a statistically significant reduction in ED compared to earlier generations.^42^, ^50^ Studies by Meyer et al^42^ and Lell et al^50^ reported that the latest generation of DSCT technology was capable of producing high resolution CT images in temporal bone scans with a smaller x‐ray current, facilitating a reduction in the ED by at least half, as shown in Figure 6. Further investigation in to the use of DSCT systems is necessary to establish the efficacy of this technology in assessing the entire facial structure following traumatic injury. Additional factors would have to be considered including financial and time costs associated with equipment and technical training.

## Conclusion

MDCT is promoted as the gold‐standard imaging modality for facial trauma, finding unparalleled support within the current literature. The most significant limitation of MDCT is the radiation burden to patients. The onus lies with radiologists and technicians to balance radiation dose and image quality, thus research into dose reduction techniques should be an ongoing process.

Radiation reduction can be achieved using ultra‐low dose MDCT with adaptive statistical IR and model‐based IR. Model‐based IR is presented as the most effective algorithm in terms of image quality, but is in its infancy with regard to facial bone imaging. More research should be conducted in a clinical context to confirm efficacy, and the technology should be developed further to include sharp and smooth kernels to maximise diagnostic value. Reducing the number of facial bone CT scans performed on each patient has also been explored. The use of MRI for pre‐operative and post‐operative scanning could mitigate accumulative dose, however time efficiency and costs would have to be considered.

Innovative methods of dose reduction include the use of intraoperative CBCT, diagnostic CBCT, and DSCT. Literature indicates that intraoperative CT can lead to improved surgical outcomes however further comparative research on doses should be undertaken. CBCT is a promising low dose modality, but has only recently been applied in non‐dental imaging. More research on the clinical value in both diagnostic and intraoperative imaging should be conducted before practical recommendation. DSCT is introduced as a future method for dose optimisation. DSCT systems reduce dose and maintain image quality, but cannot be implemented with any urgency because of the associated financial and time costs.

## Conflict of Interest

The authors declare no conflict of interest.
